# ﻿Mitogenomics, phylogeny and morphology reveal two new entomopathogenic species of *Ophiocordyceps* (Ophiocordycipitaceae, Hypocreales) from south-western China

**DOI:** 10.3897/mycokeys.109.124975

**Published:** 2024-09-26

**Authors:** Tao Sun, Yue Chen, Dong Wang, Yongdong Dai, Weiqiu Zou, Run Luo, Quanying Dong, Hong Yu

**Affiliations:** 1 Yunnan Herbal Laboratory, College of Ecology and Environmental Sciences, Yunnan University, Kunming 650504, Yunnan, China Yunnan University Kunming China; 2 The International Joint Research Center for Sustainable Utilization of Cordyceps Bioresources in China and Southeast Asia, Yunnan University, Kunming, 650504, China Yunnan University Kunming China

**Keywords:** Hepialidae, mitochondrial genome, morphology, new taxa, *Ophiocordyceps*, phylogeny

## Abstract

*Ophiocordyceps* encompasses over 300 species, demonstrating a wide range of morphological features, hosts and habitats within its species diversity. In this study, two novel species in *Ophiocordyceps* were revealed parasitising Hepialidae larva buried in soil. *Ophiocordycepsalbastroma* was morphologically characterised by white stromata, solitary and cylindrical conidiogenous cells and smooth ovoid or ellipsoidal conidia. *Ophiocordycepsnigristroma* was characterised by woody and dark brown stromata, monophialidic, swollen base and lageniform conidiogenous cells and smooth fusiform or oval conidia. The two new species formed a separate clade, respectively, based on the phylogenetic analyses of a combined dataset including nrSSU, nrLSU, *rpb1*, *rpb2*, and *tef-1α*, as well as a dataset of mitochondrial 14 protein coding genes (PCGs). They were all closely grouped with *O.sinensis*. The mitochondrial genomes of them were first reported. Their mitogenomes were all typical of circular molecules, with positive AT and GC skew, similar GC content, similar genetic composition, similar codon usage and conservative gene positions. However, the length of the mitogenomes varied. Changes in the length of the genes were the leading cause of changes in the length of mitochondrial genome of *Ophiocordyceps*. The discovery and identification of new *Ophiocordyceps* species and analysis their mitochondrial genomes may serve as foundations for phylogeny and diversity research within the genus *Ophiocordyceps*.

## ﻿Introduction

*Ophiocordyceps* was established as a genus by Petch (Petch 1931). However, the genus was not well received and was relegated to a subgenus of *Cordyceps* (Kobayasi 1941, 1982; Mains 1958). Then, the classification of *Ophiocordyceps* was emended through the morphological characteristics and molecular data and *Ophiocordyceps* was proposed as monophyletic genus within Ophiocordycipitaceae (Sung et al. 2007). The International Code of Nomenclature for Algae, Fungi and Plants was promulgated in 2012, coinciding with the incorporation of the “One fungus, one name” system. Quandt et al. (2014) suggested that the generic name, *Ophiocordyceps*, should be actively used instead of the related asexual generic names and have proposed the suppression of the anamorphic terms *Hirsutella* Pat., *Hymenostilbe* Petch, *Syngliocladium* Petch et al. and others. However, unaltered specific names of several *Hirsutella* species continue as many species have not yet been revised.

The *Ophiocordyceps* species exhibit a wide array of morphological characteristics (Mongkolsamrit et al. 2019) and has diverse hosts (Shrestha et al. 2016; Khonsanit et al. 2018; Luangsa-ard et al. 2018; Tasanathai et al. 2019, 2020; Thanakitpipattana et al. 2020; Tang et al. 2023), inhabiting various habitats such as decaying wood, leaf litter, undersides of leaves, forest plant stems and buried host insects in soil due to the varied growth environments and habits of their hosts. The global distribution of *Ophiocordyceps* species is currently most prominent in tropical and subtropical regions (Lao et al. 2021). More extensive sampling is required to gain a more comprehensive understanding of the diversity, distribution and lifestyle of species within this genus, due to the wide range of morphological features, hosts and habitats. *Ophiocordyceps* was widespread in China and many new species had recently been reported (Chen et al. 2011; Wen et al. 2013; Yang et al. 2015; Wang et al. 2018; Wang et al. 2020a; Chen et al. 2021; Sun et al. 2022; Tang et al. 2023; Fan et al. 2024).

Phylogenetic analyses of *Ophiocordyceps*, utilising datasets of multiple nuclear gene sequences, have been crucial in understanding its evolutionary relationships (Sung et al. 2007; Yang 2011; Quandt et al. 2014; Sanjuan et al. 2015; Wang et al. 2020a). Based on these analyses, *Ophiocordyceps* has been classified into four main clades: *Hirsutella*, *O.sobolifera*, *O.ravenelii* and *O.sphecocephala*. The *Hirsutella* clade can be further divided into the *H.sinensis* subclade, the *H.nodulosa* subclade, the *H.thompsonii* subclade, the *H.guyana* subclade, the *H.citriformis* subclade and the *Hirsutella* ant pathogen subclade (Sanjuan et al. 2015; Simmons et al. 2015; Wang et al. 2018). The current tally of *Ophiocordyceps* species names exceeds three hundred, as documented by the Index Fungorum (http://www.indexfungorum.org/, retrieval on 15 March 2024).

The mitochondrial genome is hereditable and its structure and composition are conservative. Additionally, it has a high copy number, low mutation rate and fast evolution rate. These characteristics made it a reliable tool for researching the origin, classification and evolution of eukaryotic species (Alexeyev et al. 2013; Aguileta et al. 2014; Williams et al. 2014). The significant difference in the fungal mitochondrial genome could be distinguished amongst different species or different individuals (Aguileta et al. 2014; Zhang et al. 2015). The size of fungal mitochondrial genome was decided by the number and length of genes, introns, intergenic regions and repeat sequences (Nie et al. 2019). Additionally, the application of phylogenetic analyses using mitochondrial protein coding genes has emerged as an innovative approach in the investigation of fungal taxonomy (Chen et al. 2021; Zhao et al. 2021; Sun et al. 2022). Therefore, it could be an approach to classification and evolution of related species by comparing fungal mitochondrial genome features (Gordon et al. 2009; Zhang et al. 2017b). Until now, more than 680 mitochondrial genomes of fungi have been published in NCBI, amongst which about 60 species belong to Hypocreales (Yang 2011; Alexeyev et al. 2013; Aguileta et al. 2014; Williams et al. 2014; Zhang et al. 2015; Zhang et al. 2017b; Nie et al. 2019).

Only about ten species of the reported mitochondrial genomes of Hypocreales belong to the family Ophiocordycipitaceae and only six are in the *Ophiocordyceps*. The mitochondrial genome of *O.sinensis* is the largest and *H.minnesotensis* has the smallest mitochondrial genome in the reported mitochondrial genomes of Ophiocordycipitaceae species (Zhang et al. 2016; Kang et al. 2017). Significant differences existed in the sizes of mitochondrial genomes of Ophiocordycipitaceae species, which were caused by the different length of intergenic regions and the different number and length of introns (Li et al. 2015; Wang et al. 2018). Many mitochondrial genomes of *Ophiocordyceps* species are in urgent need of sequencing for the purpose of studying the identification, classification, phylogeny and evolution of the species from *Ophiocordyceps*.

This study unveiled two novel species within the *Hirsutella* clade of *Ophiocordyceps* through mitogenomics, phylogeny and morphology. Furthermore, we discussed the phylogenetic relationship of *Ophiocordyceps* by conducting phylogenetic analyses based on datasets of the five target genes (nrSSU, nrLSU, *rpb1*, *rpb2* and *tef-1α*) and 14 mitochondrial protein-coding genes (PCGs), respectively. Additionally, the mitochondrial genomes of them were first reported and the features were identified. Finally, the relationships between the two new taxa and their related species were clarified by conducting phylogenetic analyses and comparing morphology and mitochondrial genome characteristics.

## ﻿Materials and methods

### ﻿Sample collection and isolation

*Ophiocordyceps* samples were collected from Lanping in Yunnan Province and Zuogong in Xizang Autonomous Region. The specimens were transferred and stored at the Yunnan Herbal Herbarium (YHH) of Yunnan University, isolated with the tissue isolating method (Liu et al. 1989; Wang et al. 2020a; Sun et al. 2022) and cultured on the culture medium (200 g potato, 20 g dextrose 20, 15–20 g agar, 10 g yeast extract, 5 g peptone in 1 litre sterile water) (Xu et al. 2019). The cultures were deposited in the Yunnan Fungal Culture Collection (YFCC) of Yunnan University.

### ﻿Morphological observations

Specimens and their habitats were photographed with a Canon 750D digital camera. The macroscopic morphological characteristics of these specimens were examined and recorded under an Olympus SZ61 microscope, including the colour and shape of stromata, the perithecial orientation and the host characteristics. The materials were cultured at 25 °C and the growth was measured every week. After 6–10 weeks, the superficial pure cultures were stuck lightly on transparent adhesive tapes, the tapes were then patched on slides and the slides were placed on Olympus CX40 and BX53 microscopes for micro-morphological observations and measurements (Wang et al. 2018; Wang et al. 2020a). At least 20 measurements were performed and the mean values were calculated excluding absolute minima and maxima.

### ﻿DNA extraction, PCR amplification and sequencing of nuclear genes of wild material

The genomic DNA was extracted from the wild material using the ZR Fungal DNA Kit (Zymo, California, USA) following manufacturer’s guidelines. The DNA extracts were checked on 1% agarose gel and the concentration and purity were detected by a NanoDrop® ND-2000 spectrophotometer (Thermo Scientific, Wilmington, USA). The five target genes, nrSSU, nrLSU, *rpb1*, *rpb2* and *tef-1α*, were amplified using the primer pairs (Vilgalys and Hester 1990; White et al. 1990; Rehner and Samuels 1994; Liu et al. 1999; Castlebury et al. 2004; Rehner and Buckley 2005; Bischoff et al. 2006; Wang et al. 2015) detailed in Suppl. material 1: table S1. The PCR mixtures contained 2 × Taq PCR Master Mix (Tiangen, Beijing, China) 25 μl, forward primer (10 μM) 0.5 μl, reverse primer (10 μM) 0.5 μl, template DNA (1 ng/μl) 1 μl and finally added sterile ddH_2_O up to 50 μl. The PCR reactions were performed in a T100 Thermal Cycler (Bio-Rad, USA) under the following conditions: denaturation at 95 °C for 4 min, 33 cycles of denaturation at 94 °C for 50s, annealing at variable temperatures for 50 s, elongation at 72 °C for 55 s and a final 8 min elongation step at 72 °C. Then the PCR products were transported to BGI genomics Co., Ltd (Chongqing, China) for sequencing.

### ﻿Extraction and sequencing of genomic DNA of pure cultures

The genomic DNA of the pure cultures was extracted using the method described above. The DNA extracts were checked on 1% agarose gel and the concentration and purity were detected by a NanoDrop® ND-2000 spectrophotometer (Thermo Scientific, Wilmington, USA). Then those were transported to BGI genomics Co., Ltd (Wuhan, China) for sequencing. The sequencing library was built by the IlluminaTruseq™ DNA Sample Preparation Kit (BGI, Shenzhen, China) and the Illumina HiSeq 4000 Platform was applied to the PE2 × 150 bp sequencing. After data quality control, the unpaired, short and low-quality reads were removed and then the clean reads were obtained (Chen et al. 2021; Zhao et al. 2021).

### ﻿Mapping nuclear genes of pure cultures

The “fungus_nr” module of GetOrganelle v.1.7.5 was employed to assembly fungus nuclear ribosomal RNA with setting ‘-R 10 -k 21,45,65,85,105’ to obtain nrSSU and nrLSU (nuclear ribosomal small and large subunits). The Sanger-sequencing three genes, containing *rpb1* and *rpb2* (the largest and second-largest subunit sequences of RNA polymerase ІІ) and *tef-1α* (the translation elongation factor 1α), of *Ophiocordyceps* were downloaded by using it as search terms in NCBI for building seed and label dataset (Suppl. material 1: table S2), then genome resequencing data were mapped through the “anonym” module with reads extending length 500 (Jin et al. 2020).

### ﻿Assembly and annotation of mitogenomes

Mitogenome reads were collected from the clean data via GetOrganelle v.1.6.2e and the mitogenomes were assembled using BLAST v.2.2.30 and SPAdes. v.3.13.0 (Jin et al. 2020; Chen et al. 2021; Zhao et al. 2021; Sun et al. 2022). The mitogenomes were initially annotated by MFannot (https://megasun.bch.umontreal.ca/RNAweasel/) and MITOS (http://mitos2.bioinf.uni-leipzig.de/index.py) (Valach et al. 2014; Jin et al. 2020; Chen et al. 2021; Zhao et al. 2021). After manual verification, the mitochondrial sequence data of the two species was uploaded to the GenBank database with the Accession Numbers: OQ658680–OQ658681.

### ﻿Sequence analyses of mitogenomes

The sequence characterisation (gene composition and nucleotide length) of the complete mitogenomes of the two species were computed using Geneious Prime^®^ v.2022.1.1 (https://www.geneious.com/) (Zhang et al. 2022). The contents of AT and GC were calculated, the start codons and the end codons of mitochondrial protein coding genes (PCGs) were detected and the codon usage was counted through PhyloSuite v.1.2.2 (Zhang et al. 2020).

### ﻿Phylogenetic analyses

For revealing the phylogenetic location and relationship of the two species and their allies, phylogenetic analyses were conducted with the homologous of five genes (nrSSU, nrLSU, *rpb1*, *rpb2* and *tef-1α*) of nuclear genes (Wang et al. 2015; Mongkolsamrit et al. 2019; Wang et al. 2020a, 2020b) and 14 PCGs (*atp6*, *atp8*, *atp9*, *cob*, *cox1*, *cox2*, *cox3*, *nad1*, *nad2*, *nad3*, *nad4*, *nad4L*, *nad5* and *nad6*) of mitogenomes (Zhang et al. 2017b; Chen et al. 2021). The Bayesian Inference (BI) and the Maximum Likelihood (ML) methods were performed for the phylogenetic analyses by MrBayes v.3.1.2 (Ronquist and Huelsenbeck 2003) and RaxML 7.0.3 (Stamatakis et al. 2008). The GTR + G + I model was determined by jModelTest v.2.1.4 (Darriba et al. 2012) with 10 million generations for the BI analysis. The ML analysis was run with the GTR + I model on 10,000 rapid bootstrap replicates. The phylogenetic relationships of *Ophiocordyceps* and related genera in the family Ophiocordycipitaceae were established using a dataset of five genes. The sequences were retrieved from GenBank. *Cordycepsmilitaris* (L.) Fr. and *C.kyusyuensis* Kawam were designated as outgroup (Suppl. material 1: table S2). *Penicilliumcitrinum* Thom and *Neurosporacrassa* Shear & B.O. Dodge were designated as outliers for another phylogenetic analysis, which was performed by combining 14 PCGs sequences concatenated from the complete mitochondrial genomes of species in the Hypocreales (Suppl. material 1: table S3).

## ﻿Results

### ﻿Phylogenetic analyses

**Figure 1.**
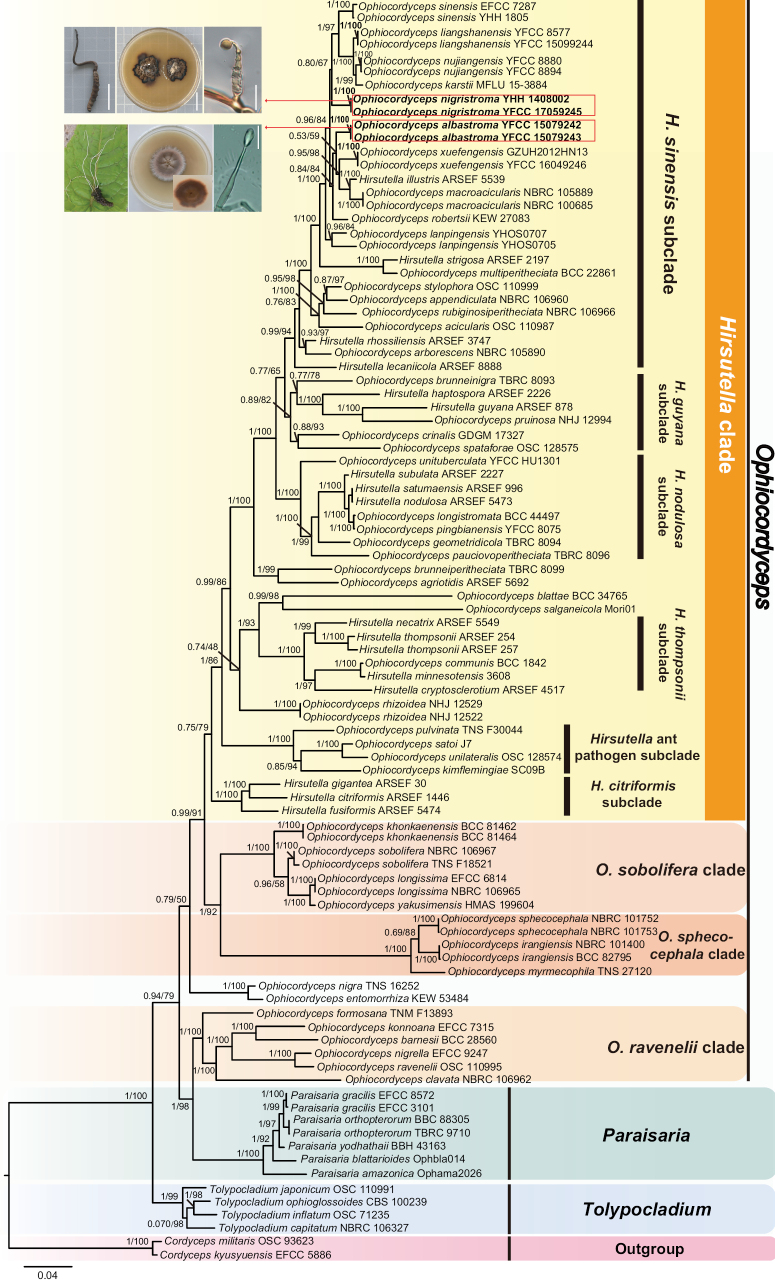
Phylogenetic relationships of *Ophiocordyceps* and related genera in the Ophiocordycipitaceae and phylogenetic placements of *O.albastroma* and *O.nigristroma.* The tree was inferred from the Bayesian Inference (BI) and the Maximum Likelihood (ML) analyses by concatenating nrSSU, nrLSU, *rpb1*, *rpb2* and *tef-1α* datasets. The BI posterior probabilities (≥ 0.5) and the ML bootstrap values (≥ 50%) were indicated at the nodes. Those new species proposed in this study are in bold type.

The Bayesian Inference (BI) and the Maximum Likelihood (ML) phylogenetic trees were built with the 94 taxa for revealing the phylogenetic relationships of *Ophiocordyceps* and related genera in the Ophiocordycipitaceae, *Cordycepsmilitaris* OSC93623 and *C.kyusyuensis* EFCC5886 were designated as outgroup (Fig. 1; Suppl. material 1: table S2). The similar topologies were obtained between the BI and ML phylogenetic analyses. The reconstructed phylogenetic tree of *Ophiocordyceps* and the related genera was similar to the analyses by Mongkolsamrit et al. (2019), *Ophiocordyceps* contained four statistically well-supported clades (namely, *Hirsutella* clade, *O.sphecocephala* clade, *O.sobolifera* clade and *O.ravenelii* clade) and the *Hirsutella* clade had six distinct subclades (namely, *H.sinensis* subclade, *H.nodulosa* subclade, *H.thompsonii* subclade, *H.guyana* subclade, *H.citriformis* subclade and *Hirsutella* ant pathogen subclade). The result was congruent with Sanjuan et al. (2015), Simmons et al. (2015) and Wang et al. (2018). All the specimens were placed into the *H.sinensis* subclade of *Hirsutella* clade.

The two specimens of *O.nigristroma* (YHH 1408001 and YFCC 17059245) clustered together and formed a separate clade (BI posterior probabilities = 1, ML bootstrap = 100%). *Ophiocordycepsnigristroma* was strongly supported to be closely related to *O.karstii*, *O.liangshanensis*, *O.nujiangensis* and *O.sinensis*. Phylogenetic analysis placed the two samples of *O.albastroma* (YFCC 15079242 and YFCC 15079243) in a separate clade (BI posterior probabilities = 1, ML bootstrap = 100%). Additionally, *O.albastroma* was closely allied to *O.xuefengensis*, *H.illustris* and *O.macroacicularis*.

As Fig. 1 shows, *O.sinensis* was clustered with *O.liangshanensis*, *O.karstii* and *O.nujiangensis* (BI posterior probabilities = 1, ML bootstrap = 97%). It indicated that *O.liangshanensis*, *O.karstii*, *O.nujiangensis* and *O.sinensis* were the most closely related. The two specimens of *O.nigristroma* formed a separate branch and clustered together with the four species (BI posterior probabilities = 0.80, ML bootstrap = 67%). While *H.illustris*, *O.albastroma*, *O.macroacicularis*, *O.robertsii* and *O.xuefengensis* were grouped together (BI posterior probabilities = 0.84, ML bootstrap = 84%), their phylogenetic relationships were more closely related. The two specimens of *O.lanpingensis* (YHOS0705 and YHOS0707) clustered in a separate clade and grouped with the species described above (BI posterior probabilities = 1, ML bootstrap = 100%). The other species in the *H.sinensis* subclade were more distantly related to the above (Fig. 1).

### ﻿Taxonomy

#### ﻿ Ophiocordyceps albastroma

Taxon classificationFungiHypocrealesOphiocordycipitaceae

Hong Yu bis, Y.D. Dai, T. Sun and Y. Chen sp. nov.

16C610DD-6998-54F7-A12C-A081E86587C4

847670

Fig. 2

##### Etymology.

The epithet ‘*albastroma*’ refers to white stromata this species.

##### Holotype.

China, Yunnan Province, Nujiang Lisu Autonomous Prefecture, Lanping County, the Bailongtan Mountains (26°37'N, 99°37'E, alt. 3200 m), isolated from the Hepialidae, 26 July 2016, Hong Yu, (***holotype***: YHH 1507001; ***ex*-*type living culture***: YFCC 15079242).

##### Description.

***Teleomorph***: Stromata arising from Hepialidae larva buried in soil, slender, solitary or gregarious, unbranched, 5.1–11.8 cm long, 0.08–0.1 cm wide at the base and 0.02–0.03 cm wide at the top. The morphology of perithecia and asci was not observed, as the collections did not include any specimens that had reached sexual maturity.

***Anamorph***: *Hirsutella*-type anamorph. Colonies on PDA slow-growing, attaining a diameter of 13–15 mm after 30 days at 20 °C. Colonies pale yellow, high mycelial density, felty, texture hard, microtomentum, white margin, with star ray folds; reverse pale brown. Hyphae hyaline, branched, smooth-walled, 1.4–2.3 μm wide. Phialides from aerial mycelium straight to slightly ﬂexuose, solitary, cylindrical, usually with a slightly swollen basal part, tapering into the apex form a long neck, 6.5–21.3 × 0.4–1.3 μm, 0.6–1.6 µm wide at the base and 0.2–0.7 µm wide at the apex. Conidia usually one-celled, occasionally two-celled, hyaline, smooth, ovoid to ellipsoidal, 2.2–3.6 × 1.1–1.9 µm, conidia secrete mucus.

**Figure 2.**
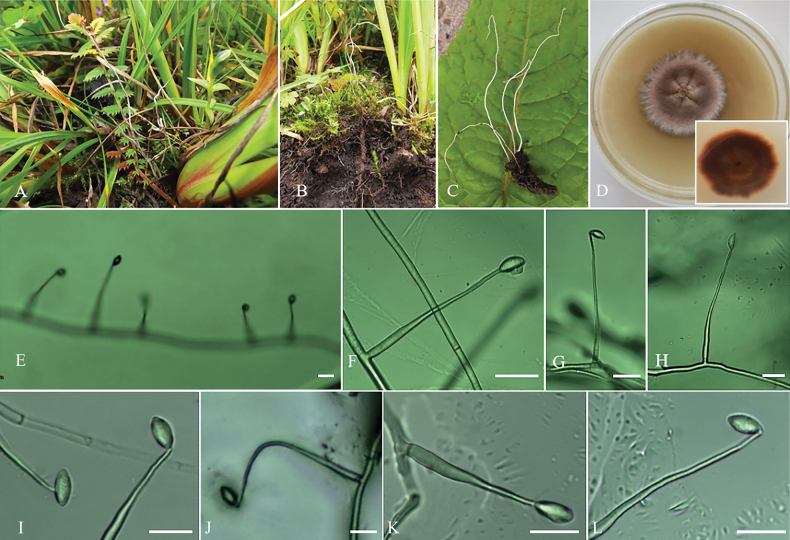
*Ophiocordycepsalbastroma***A–C** specimen in the field **D** colony obverse and colony reverse on PDA with peptone and yeast extract powder **E–L** conidiogenous cells and conidia. Scale bars: 4 µm (**E–L**).

***Host***: Larvae of Hepialidae.

##### Habitat.

Parasitic on Hepialidae larvae in the alpine soil amongst the *Iris* spp.

##### Distribution.

The Bailongtan Mountains, Lanping County, Nujiang Lisu Autonomous Prefecture, Yunnan Province, China.

##### Additional specimens examined.

China, Yunnan Province, Nujiang Lisu Autonomous Prefecture, Lanping County, The Bailongtan Mountains (26°37'N, 99°37'E, alt. 3200 m), isolated from the Hepialidae, 26 July 2016, Hong Yu, dried culture on PDA (paratype YHH 1507002, ex-paratype living culture YFCC 15079243).

##### Notes.

*Ophiocordycepsalbastroma* was closely phylogenetically related to *H.illustris*, *O.macroacicularis* and *O.xuefengensis*. The morphological characteristic common to all four species was the formation of stromata on the head of the host. However, the length of the stromata varies, *O.albastroma* had a stromata length longer than *O.macroacicularis*, but shorter than *O.xuefengensis* (Table 1). The phialides of *O.albastroma* was smaller than that of *H.illustris* and *O.albastroma* had a conidia length much shorter than *H.illustris*. Moreover, the greatest peculiarity of *O.albastroma* was the white stromata. Thus, the morphological features and molecular phylogenetic results supported that *O.albastroma* was a new species in the genus *Ophiocordyceps*.

**Table 1.** Morphological comparison of *Ophiocordycepsalbastroma*, *O.nigristroma* and their allies.

Species	Host	Stromata	Ascomata	Asci	Ascospores	Phialides	Conidia	Reference
*Hirsutellaillustris*	–	–	Superficial	–	–	Soliary, 50–100 μm long, subcylindrical, 7 μm wider,	Ellipsoidal, 15–20 μm	Minter and Brady (1980)
*Ophiocordycepsalbastroma*	Hepialidae larvae	Several or solitary, 51–118 mm	–	–	–	Solitary, cylindrical, 6.5–21.3 × 0.4–1.3 μm	Ovoid to ellipsoidal, 2.2–3.6 × 1.1–1.9 µm	This study
*O.karstii*	On dead larva of *Hepialusjianchuanensis*	140–145 × 2–4 mm	Superficial, flask-shaped, 600–765 × 247–323 μm	Narrow cylindrical, 186–228 × 8–12 μm	Fusiform, 173–202 × 3–5 μm, not breaking into secondly spores	–	–	Li et al. (2016)
*O.lanpingensis*	Hepialidae larva	Several or solitary, 50–160 × 0.2–1.3 mm	Superficial, ovoid, 310–370 × 200–240 μm	Cylindrical, 240–300 × 5.1–6.5 μm	Cylindrical, 240–300 × 1.4 μm; not fragmenting into part-spore, multiseptate with indistinct septation, 3.3–4.9 × 1.1–1.4 μm	–	–	Chen et al. (2013)
*O.liangshanensis*	Hepialidae larvae	Cylindrical, 200–300 × 1.5–2.5 mm	Superficial, long ovoid, 450–740 × 300–450 μm	Cylindrical, 260–480 × 8–12 μm	Fasciculate, thread-like, slender and long, 170–240 × 2.5–4.1 μm	Monophialidic, 46.9–75.6 μm long, subcylindrical, 3.8–4.7 μm basal wide	Ellipsoid, citriform or shape of an orange segment, 8.0–12.6 × 3.6–5.0 μm	Wang et al. (2021)
*O.macroacicularis*	Lepidoptera larva	2–5 stromata occurred from host, 100–170 × 1.3–2.5 (130 × 1.5) mm	Superficial, ovoid, 410–760 × 260–420 (534.8 × 333.3) μm	235–310 (265.7)	Not divided	–	Oval to lemon-shaped	Ban et al. (2014)
*O.nigristroma*	Hepialidae larva	84–136 mm	–	–	–	Solitary, cylindrical, 19.2–32.7 × 3.0–6.6 μm	Fusiform or oval, 5.0–9.5 × 3.6–6.9 µm	This study
*O.nujiangensis*	Hepialidae larvae	Solitary, 148–182 mm long	–	–	–	54.9–76.5 µm long, base width 3.6–4.9 µm, tip width 1.0–1.5 µm	Oval or fusiform, 6.4–11.2 × 3.7–6.4 µm	Sun et al. (2022)
*O.robertsii*	Hepialidae larva	Single, cylindrical, 100–380 × 3–4 mm	Superficial, elongate-obvate or elliptical, 600–880 × 300–400 μm	Narrowly cylindrical, 280–400 × 9–10 μm	Filiform, multiseptate, 280 × 3 μm, breaking into secondary ascospores, 5–6 × 3 μm	–	–	Cunningham (1921)
*O.sinensis*	Hepialidae larva	Single, occasionally 2–3, 40–110 mm	Nearly superficial, ellipsoidal to ovate, 380–550 × 140–240 μm	Slender, long, 240–485 × 12–16 μm	Usually 2–4 mature ascospores, multiseptate, not breaking into secondary ascospores, 160–470 × 5–6 μm	–	–	Liang (2007)
*O.xuefengensis*	Hepialidae larva	1–4 arising from head or other parts of host, cylindrical, 140–460 × 2–7 mm	Superficial, long ovoid, 416–625 × 161–318 μm	Cylindrical, 191–392 × 4.5–8.9 μm	Thread-like, with many septa, not breaking into secondary ascospores, 130–380 × 1.4–5.2 μm	–	–	Wen et al. (2013)

#### ﻿ Ophiocordyceps nigristroma

Taxon classificationFungiHypocrealesOphiocordycipitaceae

Hong Yu bis, T. Sun, W.Q. Zou and Y.D. Dai sp. nov.

7B71048D-3B4A-5A19-8B06-E06242F62F6A

847630

Fig. 3

##### Etymology.

The epithet ‘*nigristroma*’ refers to black stromata produced.

##### Type.

China, Xizang Autonomous Region, Changdu City, Zuogong County, the Dongda Mountains (29°43'N, 98°01'E, alt. 4963 m), isolated from Hepialidae larva, 2 June 2017, Hong Yu, (***holotype***: YHH 1705001; ***ex*-*type living culture***: YFCC 17059245).

##### Description.

***Teleomorph***: Stromata grew from the head of the host Hepialidae larva buried in soil, sturdy, solitary, unbranched, woody, hard, dark brown, 8.4–13.6 cm long, 0.25–0.45 cm wide at the base and 0.1–0.2 cm wide at the top. The morphology of perithecia and asci was not observed, as the collections did not include any specimens that had reached sexual maturity.

**Figure 3.**
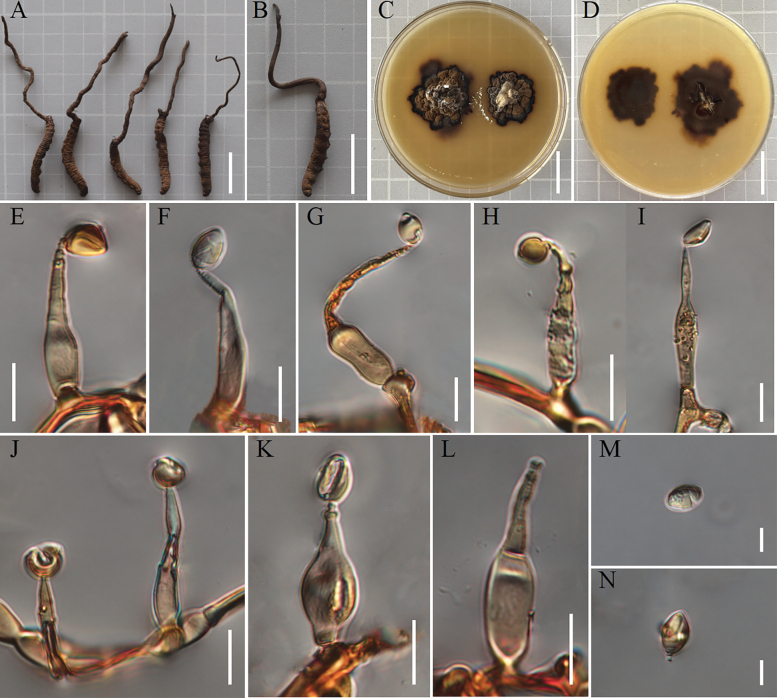
*Ophiocordycepsnigristroma***A, B** intact wild material **C** colony obverse on PDA with peptone and yeast extract powder **D** colony reverse on PDA with peptone and yeast extract powder **E–K** conidiogenous cells and conidia **L** conidiogenous cells **M, N** conidia. Scale bars: 2 cm (**A–D**); 10 µm (**E–L**); 5 µm (**M–N**).

***Anamorph***: *Hirsutella*-type anamorph. Colonies on PDA slow-growing, attaining a diameter of 18–21 mm after 14 weeks at 20 °C. Colonies dark brown to black, high mycelial density, texture hard, reverse dark brown. Hyphae hyaline, smooth-walled, 1.6–2.7 μm wide. Phialides from aerial mycelium straight to slightly ﬂexuose, monophialidic, smooth, swollen base, lageniform, tapering into the apex forming a neck, 19.2–32.7 × 3.0–6.6 μm and 0.5–1.8 µm wide at the apex. Conidia one-celled, hyaline, smooth, fusiform or oval, 5.0–9.5 × 3.6–6.9 µm.

##### Additional specimens examined.

China, Xizang Autonomous Region, Changdu City, Zuogong County, the Dongda Mountains (29°43'N, 98°01'E, alt. 4963 m), isolated from the Hepialidae, 1 August 2014, Hong Yu (paratype YHH 1408001).

##### Known distribution.

At present known only from China.

##### Notes.

*Ophiocordycepsnigristroma* was closely phylogenetically related to *O.karstii*, *O.liangshanensis*, *O.nujiangensis* and *O.sinensis*. The formation of stromata on the head of the host was a characteristic common to all four species. However, the length of the stromata varies amongst the four species. *Ophiocordycepsnigristroma* had a stromata length slightly longer than *O.sinensis*, but shorter than *O.karstii*, *O.liangshanensis* and *O.nujiangensis*. *O.nigristroma*, on the other hand, had shorter phialides than *O.liangshanensis* and *O.nujiangensis* (Table 1). Thus, the morphological characteristics and molecular phylogenetic results supported that *O.nigristroma* was a new species in the genus *Ophiocordyceps*.

### ﻿Mitochondrial genome features analysis

#### ﻿The two mitochondrial genomes organisation and gene content

**Figure 4.**
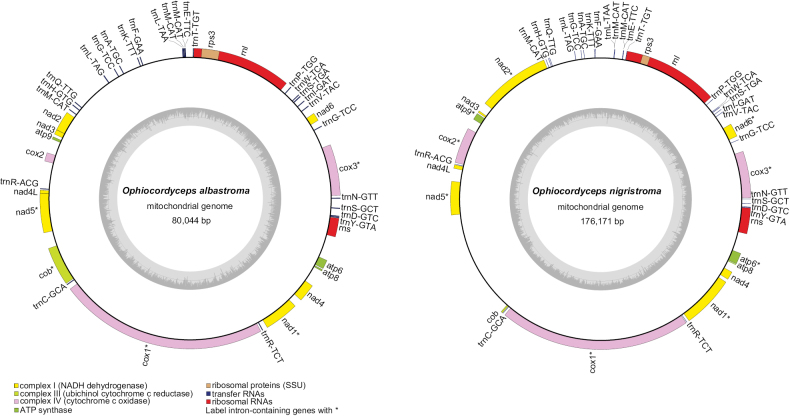
Circular maps of the newly-sequenced mitogenomes of *Ophiocordycepsalbastroma and O.nigristroma*. Genes are represented with different colour blocks.

As Fig. 4 shows, the complete mitochondrial genomes of *Ophiocordycepsalbastroma* and *O.nigristroma* were typical of circular molecules; however, their lengths were different. The GC content of the two species showed comparable values (30.9% and 30.2%). The two mitogenomes were all positive AT and GC skew. Their genic regions accounting for 66.01% and 63.52%, respectively, containing 15 protein coding genes (PCGs), 2 rRNA genes and 26 tRNA genes.

Fifteen PCGs were annotated in the mitochondrial genomes of *Ophiocordycepsalbastroma* and *O.nigristroma*, containing three ATP synthase subunit genes (*atp6*, *atp8* and *atp9*), one cytochrome b gene (*cob*), three cytochrome c oxidase subunit genes (*cox1*, *cox2* and *cox3*), seven NADH dehydrogenase complex subunit genes (*nad1*, *nad2*, *nad3*, *nad4*, *nad5*, *nad6* and *nad4L*) and one ribosomal protein S3 gene (*rps3*). The *cox1* gene was the longest amongst all 15 genes of the four mitogenomes, ranging from 18,039 to 36,379 bp in length (including intronic regions). When the length of the intronic region was subtracted, the length of the *nad5* gene was the longest and the *atp8* gene is the shortest, at 147 bp (Suppl. material 1: tables S4, S5). The PCGs usually used NTG (ATG and GTG) as the start codon and TAN (TAG and TAA) as the end codon; however, in the mitochondrial genome of *O.nigristroma*, TTA was the start codon of *cob* (Suppl. material 1: tables S4, S5).

Each of the two mitogenomes involved two rRNA genes: the large subunit ribosomal RNA gene (*rnl*) and the small subunit ribosomal RNA gene (*rns*), with the total length of rRNA genes altering from 10,170 bp to 23,0042 bp (Suppl. material 1: tables S4, S5). Additionally, the *rps3* gene was embedded in the region of the *rnl* gene. A total of 26 tRNA genes were identified in the mitogenomes of *O.albastroma* and *O.nigristroma*. All tRNA genes ranged in length from 71 to 86 bp and encoded all 20 amino acids in the two mitogenome (Suppl. material 1: tables S4, S5).

A total of 39 and 41 intergenic regions, accounting for 36.78% and 33.99% respectively, were found from the mitogenomes of *O.albastroma* and *O.nigristroma* (Suppl. material 1: tables S4, S5). The longest intergenic region between the two mitogenomes was between *nad5* and *cob* in the *O.nigristroma* mitogenome which was 20,716 bp long. The two mitochondrial genomes exhibit minimal overlapping ranges (Suppl. material 1: tables S4, S5). The only 1 bp gene overlap was between *nad4L* and *nad5*, observed from the mitogenome of *O.albastroma*.

#### ﻿Codon usage and the frequency of amino acid usage in protein-coding genes

**Figure 5.**
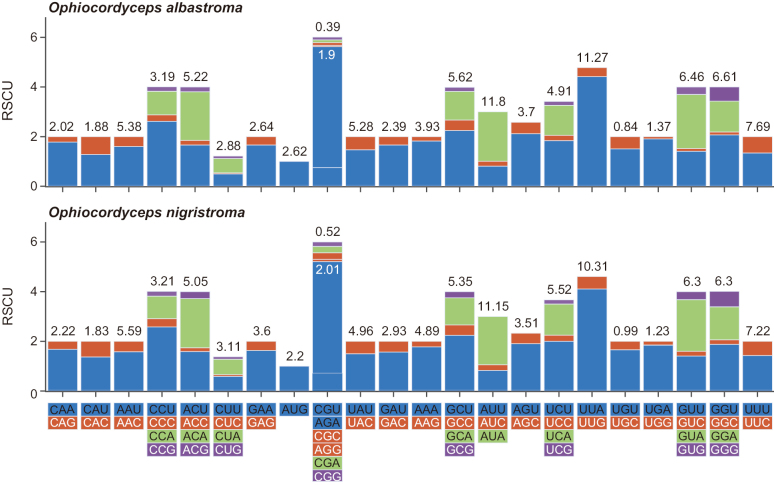
Codon usage analysis and the frequency of amino acid usage in the PCGs of the complete mitochondrial genome of *Ophiocordycepsalbastroma* and *O.nigristroma*. The histograms show the RSCU values and the colours represent the codons. The figures above the histograms are the frequency of amino acid usage.

In the PCGs of the mitochondrial genomes of the two species, the frequency of amino acid usage varied, but the trend remained the same. Ile had the highest frequency, followed by Leu2, Val and Gly. Cys was used the least (Fig. 5; Suppl. material 1: tables S6, S7). The numbers of codons used were 4889 and 5363, respectively, in the PCGs of mitochondrial genomes of *Ophiocordycepsalbastroma* and *O.nigristroma*. As Fig. 5 shows, UUA, AUA, UUU and AAU were used most frequently. In terms of relative synonymous codon usage (RSCU), the preferred codons used were AGA (for Arg), UUA (for Leu2), CCU (for Pro) and GCU (for Ala).

### ﻿Phylogenetic analyses based on mitochondrial protein-coding genes

**Figure 6.**
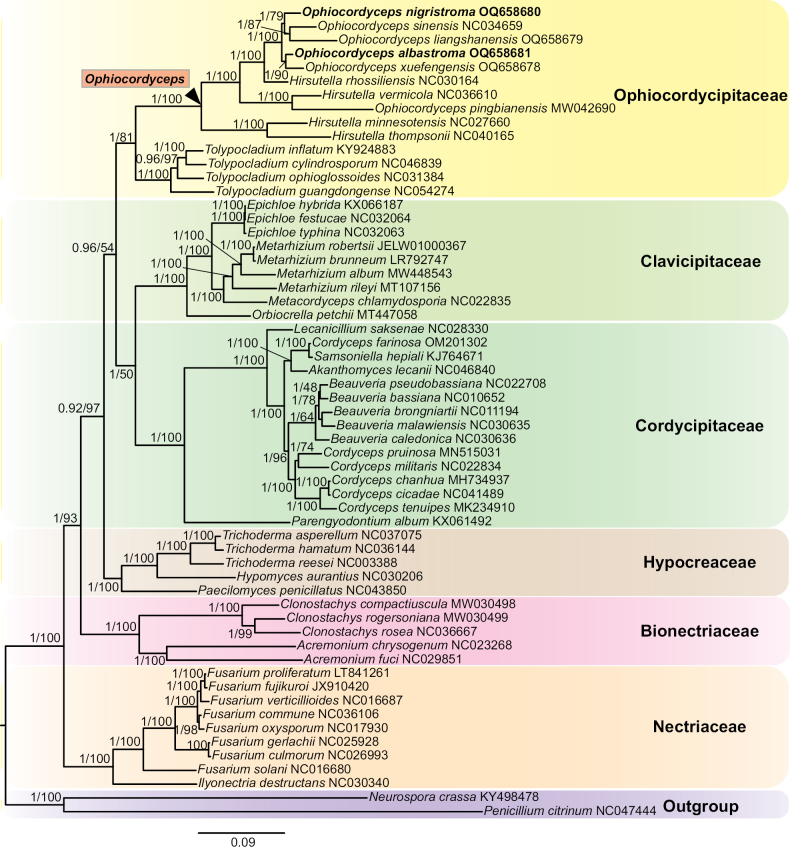
Phylogenetic tree of Hypocreales, based on the Bayesian Inference (BI) and the Maximum Likelihood (ML) analyses of 14 mitochondrial protein coding genes (PCGs). The 14 PCGs included *atp6*, *atp8*, *atp9*, *cob*, *cox1*, *cox2*, *cox3*, *nad1*, *nad2*, *nad3*, *nad4*, *nad4L*, *nad5* and *nad6*. Values at the nodes were the BI posterior probabilities and the ML bootstrap proportions, respectively. The specimen analysed in this study is in bold type.

The 14 PCGs of the two mitogenomes were chosen for phylogenetic analyses, including *atp6*, *atp6*, *atp9*, *cob*, *cox1*, *cox2* and *cox3*, *nad1*, *nad2*, *nad3*, *nad4*, *nad4L*, *nad5* and *nad6*. The BI and the ML trees were estimated for phylogenetic analyses of Hypocreales, based on the mitochondrial PCGs dataset of 59 species. *Penicilliumcitrinum* and *Neurosporacrassa* were designated as outgroup (Suppl. material 1: table S3). As shown in Fig. 6, the topology was like the analyses by Chen et al. (2021) and Zhao et al. (2021), six well-supported clades being recognised in Hypocreales, namely Bionectriaceae, Clavicipitaceae, Cordycipitaceae, Hypocreaceae, Nectriaceae, and Ophiocordycipitaceae. The specimens of the two species (*Ophiocordycepsalbastroma* and *O.nigristroma*) were clustered together with *H.minnesotensis*, *H.rhossiliensis*, *H.thompsonii*, *H.vermicola*, *O.liangshanensis*, *O.pingbianensis*, *O.xuefengensis* and *O.sinensis* in *Ophiocordyceps*. They formed a separate clade with 100% statistical support, respectively and were also closely grouped with *O.sinensis* (Fig. 6).

### ﻿Comparative analysis of the mitochondrial genomes of *Ophiocordyceps*

#### ﻿Feature comparison of mitochondrial genomes

The mitochondrial genomes of *H.minnesotensis*, *H.rhossiliensis*, *H.thompsonii*, *H.vermicola*, *O.albastroma*, *O.lanpingensis*, *O.liangshanensis*, O. *pingbianensis*, *O.nigristroma*, *O.sinensis* and *O.xuefengensis* were 52,267–226,206 bp in length (Table 2). This suggested that the differences in the full length of mitochondrial genomes amongst the *Ophiocordyceps* species were highly variable. The mitochondrial genome of *O.liangshanensis* had a full length of 226,206 bp, making it the longest, while *H.minnesotensis* had the shortest mitogenome length. The full length of the mitochondrial genome of *O.sinensis* was the longest amongst *Ophiocordyceps* species in the previous research (Chen et al. 2021; Bibi et al. 2023). However, the results of this study showed that both *O.liangshanensis* and *O.nigristroma* had longer mitogenome lengths than *O.sinensis*.

**Table 2.** The mitochondrial genomes feature of *Ophiocordyceps* species.

Organism	Full length (bp)	Coding region length (bp)	No. of PCGs	No. of rR NA	No. of tRNA	Non-coding region ratio	A+T (%)	AT skew	GC skew
*Hirsutellaminnesotensis*	52,267	20,486	15	2	25	0.28	71.6	0.011	0.099
*H.rhossiliensis*	62,516	23,776	15	2	26	0.45	71.8	0.016	0.090
*H.thompsonii*	62,548	21,389	14	2	27	0.32	70.2	0.018	0.090
*H.vermicola*	53,836	20,345	14	2	25	0.33	74.7	-**0.005**	0.122
*O.albastroma*	80,044	25,283	15	2	26	0.36	69.8	0.038	0.085
*O.lanpingensis*	117,564	20,940	15	2	20	0.19	68.9	0.052	0.082
*O.liangshanensis*	226,206	55,222	15	2	25	0.20	69.4	0.060	0.090
*O.nigristroma*	176,171	39,650	15	2	26	0.34	69.1	0.059	0.082
*O.pingbianensis*	80,401	18,885	15	2	25	0.33	70.1	0.028	0.086
*O.sinensis*	157,566	21,896	15	2	27	0.18	69.8	0.050	0.094
*O.xuefengensis*	78,763	17,437	15	0	26	0.42	70.1	0.030	0.091

As Table 2 shows, the mitochondrial genome of *O.sinensis* was the most compact, while that of *H.rhossiliensis* was the loosest. Except for the mitochondrial genomes of *H.thompsonii* and *H.vermicola*, which were not annotated with the *rps3* gene and contained a total of 14 PCGs, the remaining genomes contained 15 PCGs. The mitochondrial genomes of all species, except *O.xuefengensis*, contained two rRNA genes, *rnl* and *rns.* The number of tRNA in the mitochondrial genomes of the 11 species ranged from 20 to 27. *H.vermicola* had the highest proportion of AT content in the mitochondrial genome at 74.7%. The mitochondrial genome with the highest proportion of GC content was that of *O.nigristroma*, with a ratio of 30.9%. Apart from *H.vermicola*, the remaining 10 species have positive AT skew in their mitochondrial genomes. The GC skew of the mitochondrial genome was positive in all 11 species.

#### ﻿Gene arrangement analysis

**Figure 7.**
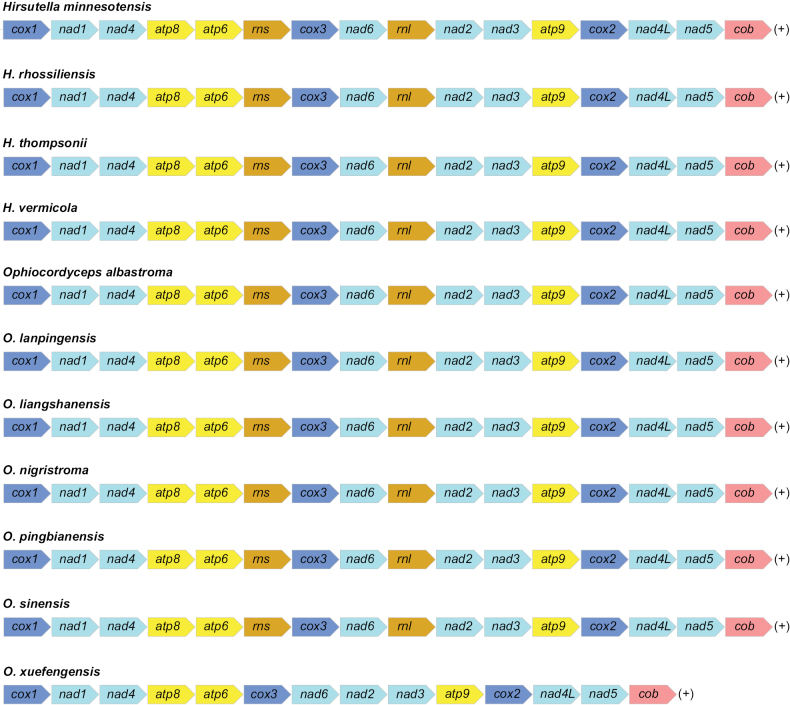
A comparison of gene order amongst the *Ophiocordyceps* mitogenomes. Genes are represented with different colour blocks.

The positions of the genes were conservative across the mitochondrial genomes of the *Ophiocordyceps* species (Fig. 7). Except the positions of the *rns* and *rnl* genes were absent in *O.xuefengensis*, the genetic order was consistent amongst the mitogenomes.

## ﻿Discussion

*Ophiocordyceps* is the largest genus in the family of Ophiocordycipitaceae, with four clades (the clade of *Hirsutella*, the clade of *O.ravenelii*, the clade of *O.sobolifera* and the clade of *O.sphecocephala*) (Sung et al. 2007; Quandt et al. 2014). The clade of *Hirsutella* is usually divided into six subclades, which are the subclade of *H.citriformis*, the subclade of *H.guyana*, the subclade of *H.nodulosa*, the subclade of *H.sinensis*, the subclade of *H.thompsonii* and the subclade of *Hirsutella* ant pathogen (Sanjuan et al. 2015; Simmons et al. 2015; Wang et al. 2018).

The subclade of *H.sinensis* contains a variety of species, of which *O.sinensis* is the best known for its medicinal properties (Han et al. 2019; Li et al. 2021). Dai et al. (2024) have described *O.sinensis* and its close relatives as the *O.sinensis*- species complex lineage. They believe that the phylogenetic relationships within this clade may be a valuable key to unlocking new understanding of speciation, adaptation and origin of functional component, due to the complex’s uniqueness and specificity (Dai et al. 2020, 2024). We described the new species *O.albastroma* and *O.nigristroma*, which are closely related species to *O.sinensis*. These two species belong to the *O.sinensis*- species complex lineage. They share many similar features, such as the fact that their stromata are all formed on the head of the host Hepialidae larva. The two new species clustered in the subclade of *H.sinensis*, based on the phylogenetic analysis of the combined datasets of nrSSU, nrLSU, *rpb1*, *rpb2* and *tef-1α* and mitochondrial 14 protein-coding genes (PCGs) datasets. However, they possess many peculiarities which make them a separate species. *Ophiocordycepsalbastroma* was morphologically characterised by solitary or gregarious, unbranched and white stromata, branched and smooth-walled hyphae, solitary and cylindrical conidiogenous cells from aerial mycelium straight to slightly ﬂexuose and smooth ovoid or ellipsoidal conidia. *Ophiocordycepsnigristroma* was morphologically characterised by solitary, woody and dark brown stromata, smooth-walled hyphae, monophialidic, swollen baseand lageniform conidiogenous cells and smooth fusiform or oval conidia.

Phylogenetic analyses showed that the reconstructed phylogenetic framework of *Ophiocordyceps* was consistent with previous studies (Sanjuan et al. 2015; Simmons et al. 2015; Wang et al. 2018; Mongkolsamrit et al. 2019). *O.albastroma* was grouped phylogenetically with *O.xuefengensis*, *H.illustris* and *O.macroacicularis*. Nevertheless, there was an obvious distinction between them in their morphological characteristics, especially in the length of the stromata. *O.nigristroma* was closely phylogenetically related to *O.sinensis*, *O.liangshanensis* and *O.karstii*. Additionally, the length of the stromata varies between the four species. The two new species clustered into the different clades and the two clades formed sister groups, indicating that they were closely related. However, by comparing their morphological features, it was found that there was a difference in the number of stromata between the sister groups. The stromata of the clade with *O.albastroma* were several or solitary, but the stromata of the clade with *O.nigristroma* were solitary. They also had a number of micromorphological differences, which, however, were difficult to compare due to the uncertain maturation times of these species in the wild and the difficulty of isolating and cultivating strains. The lack of descriptions of sexual or asexual morphological features (Table 1) results that it is difficult to make a comprehensive comparison.

The phylogenetic analysis of mitochondrial genes became an adequate means to delimit fungal species (Nie et al. 2019; Meng et al. 2020). More importantly, it is able to classify and distinguish closely-related species by comparing fungal mitochondrial genome features (Gordon et al. 2009; Zhang et al. 2015, 2017b). In this study, the two novel mitogenomes of *O.albastroma* and *O.nigristroma* were reported, their characteristics were identified and the mitogenomes of the genus were compared. The phylogenetic tree of Hypocreales, based on the Bayesian Inference (BI) and the Maximum Likelihood (ML) analyses of 14 PCGs showed that the two species and *O.sinensis* clustered together in a separate clade. The two species were closely-related species. Their mitogenomes were all typical of circular molecules, with positive AT and GC skew, similar GC content, similar genetic composition, similar codon usage and conservative gene positions. However, the length of the two mitogenomes varied considerably. The mitochondrial genomes of *O.nigristroma*, which was more closely related to *O.sinensis*, was larger than that of *O.albastroma*, species in their sister clade.

Changes in the length of the genes such as *rnl* and *rns* were the leading cause of changes in the length of mitochondrial genome of *Ophiocordyceps*. Variations between these mitochondrial genomes effectively distinguish the related species of *O.sinensis* and their phylogenetic positions were determined by the BI and ML analysis of mitochondrial PCGs. Apart from *Ophiocordyceps* species, the characteristics of mitochondrial genome are also valuable for species classification and phylogenetic analysis in other organisms. By studying the four mitogenomes of *Clonostachys*, differences amongst these mitogenomes were identified and a potential new species was discovered (Zhao et al. 2021). Comparing mitochondrial genomes might be a suitable approach to resolve taxonomic and phylogenetic issues amongst related species, particularly when certain morphological features are unobservable. The mitochondrial genome has gradually come to the attention of researchers as second-sequencing sequencing techniques have evolved, as the mitogenomes were important for taxonomic, phylogenetic and evolutionary research.

*Ophiocordyceps* encompasses more than 300 species names (http://www.indexfungorum.org/, retrieved on 15 March 15 2024), demonstrating a wide range of morphological features, hosts and habitats within its species diversity. To further investigate the systematic evolution and diversity of this genus, it is imperative to gather a broader range of samples and identify additional new species. Additionally, a comparative analysis of *Ophiocordyceps* mitogenomes could be an important foundation for phylogenetic and evolutionary studies of *Ophiocordyceps*. We will devote our efforts to studying the mitochondrial genome of the genus so that the mitogenomes can be applied to further resolve the taxonomic, phylogenetic and evolutionary status of *Ophiocordyceps*.

## Supplementary Material

XML Treatment for Ophiocordyceps albastroma

XML Treatment for Ophiocordyceps nigristroma

## ﻿Additional information

### Author ORCIDs

Tao Sun https://orcid.org/0000-0001-7837-2101

Yue Chen https://orcid.org/0009-0008-1330-4026

Hong Yu https://orcid.org/0000-0002-2149-5714

### Data availability

All sequence data generated for this work can be accessed via GenBank: https://www.ncbi.nlm.nih.gov/genbank/.

## Supplementary materials

Supplementary material 1 Supplementary information Data type zip Explanation note **table S1.** The primer information of each gene fragment used for DNA amplification in this study. **table S2.** Relevant species information and GeneBank accession numbers for phylogenetic research in this study. **table S3.** The information of species and their mitochondrial genomes for constructing the mitochondrial-genome phylogenetic tree of Hypocreales. **table S4.** Gene component of the mitogenome from *Ophiocordycepsalbastroma.***table S5.** Gene component of the mitogenome from *Ophiocordycepsnigristroma.***table S6.** Codon usage and the frequencies of amino acids in the PCGs of the complete mitochondrial genome of *Ophiocordycepsalbastroma.***table S7.** Codon usage and the frequencies of amino acids in the PCGs of the complete mitochondrial genome of *Ophiocordycepsnigristroma*. https://binary.pensoft.net/file/1142391This dataset is made available under the Open Database License (http://opendatacommons.org/licenses/odbl/1.0/). The Open Database License (ODbL) is a license agreement intended to allow users to freely share, modify, and use this Dataset while maintaining this same freedom for others, provided that the original source and author(s) are credited.Tao Sun, Yue Chen, Dong Wang, Yongdong Dai, Weiqiu Zou, Run Luo, Quanying Dong, Hong Yu
