# Supplementary material for: ﻿Mitogenomics, phylogeny and morphology reveal two new entomopathogenic species of Ophiocordyceps (Ophiocordycipitaceae, Hypocreales) from south-western China
Source: MycoKeys. 2024 Sep 26;109:49–72. doi: 10.3897/mycokeys.109.124975 (PMC11450462; doi:10.3897/mycokeys.109.124975)
Supplement: Supplementary material 1 — Supplementary information [file mycokeys-109-049-s001.zip › revised Supplementary Files/Table S1.docx]

**Table S1 The primer information of each gene fragment used for DNA amplification in this study**

| **Gene** | **Primer name** | **Primer sequence (5ʹ-3ʹ)** | **Reference** |
| --- | --- | --- | --- |
| nr*SSU* | CoF | TCTCAAAGATTAAGCCATGC | Wang et al. 2015 |
|  | CoR | TCACCAACGGAGACCTTG |  |
| nr*LSU* | LR5 | ATCCTGAGGGAAACTTC | Vilgalys and Hester 1990;  Rehner and Samuels 1994 |
|  | LR0R | GTACCCGCTGAACTTAAGC |  |
| *tef-1α* | 983F | GCYCCYGGHCAYCGTGAYTTYAT | Rehner and Buckley 2005 |
|  | 2218R | ATGACACCRACRGCRACRGTYTG |  |
| *rpb1* | CRPB1A | CAYCCWGGYTTYATCAAGAA | Castlebury et al. 2004;  Bischoff et al. 2006 |
|  | RPB1C | CCNGCDATNTCRTTRTCCATRTA |  |
| *rpb2* | fRPB2-5F | GAYGAYMGWGATCAYTTYGG | Liu et al. 1999 |
|  | fRPB2-7cR | CCCATRGCTTGYTTRCCCAT |  |
| ITS | ITS4 | TCCTCCGCTTATTGATATGC | White et al. 1990 |
|  | ITS5 | GGAAGTAAAAGTCGTAACAAGG |  |
