# Supplementary material for: ﻿Mitogenomics, phylogeny and morphology reveal two new entomopathogenic species of Ophiocordyceps (Ophiocordycipitaceae, Hypocreales) from south-western China
Source: MycoKeys. 2024 Sep 26;109:49–72. doi: 10.3897/mycokeys.109.124975 (PMC11450462; doi:10.3897/mycokeys.109.124975)
Supplement: Supplementary material 1 — Supplementary information [file mycokeys-109-049-s001.zip › revised Supplementary Files/Table S2.docx]

**Table S2 Relevant species information and GeneBank accession numbers for phylogenetic research in this study**

| **Species** | **Voucher information** | **Host/Substratum** | **GenBank accession number** | | | | |
| --- | --- | --- | --- | --- | --- | --- | --- |
|  |  |  | nr**SSU** | nr**LSU** | ***tef1-a*** | ***rpb1*** | ***rpb2*** |
| *Cordyceps militaris* | OSC 93623 | Lepidoptera | AY184977 | AY184966 | DQ522332 | DQ522377 |  |
| Cordyceps kyusyuensis | EFCC 5886 | Lepidoptera | EF468960 | EF468813 | EF468754 | EF468863 | EF468917 |
| *Hirsutella citriformis* | ARSEF 1446 | Hemiptera: Cixiidae | KM652065 | KM652106 | KM651990 | KM652031 |  |
| *Hirsutella cryptosclerotium* | ARSEF 4517 | Hemiptera: Pseudococcidae | KM652066 | KM652109 | KM651992 | KM652032 |  |
| *Hirsutella fusiformis* | ARSEF 5474 | Coleoptera: Curculionidae | KM652067 | KM652110 | KM651993 | KM652033 |  |
| *Hirsutella gigantea* | ARSEF 30 | Hymenoptera: Pamphiliidae |  | JX566977 | JX566980 | KM652034 |  |
| *Hirsutella guyana* | ARSEF 878 | Hemiptera: Cicadellidae | KM652068 | KM652111 | KM651994 | KM652035 |  |
| *Hirsutella haptospora* | ARSEF 2226 | Acari: Uropodina |  |  | KM651995 | KM652036 |  |
| *Hirsutella illustris* | ARSEF 5539 | Hemiptera: Aphididae | KM652069 | KM652112 | KM651996 | KM652037 |  |
| *Hirsutella lecaniicola* | ARSEF 8888 | Hemiptera: Coccidae | KM652071 | KM652114 | KM651998 | KM652038 |  |
| *Hirsutella minnesotensis* | 3608 | Nematoda | JPUM01000376 | JPUM01000376 | JPUM01000211 | JPUM01000139 | JPUM01000138 |
| *Hirsutella necatrix* | ARSEF 5549 | Acari | KM652073 | KM652116 | KM651999 | KM652039 |  |
| *Hirsutella nodulosa* | ARSEF 5473 | Lepidoptera: Pyralidae | KM652074 | KM652117 | KM652000 | KM652040 |  |
| *Hirsutella rhossiliensis* | ARSEF 3747 | Tylenchida: Criconematidae | KM652080 | KM652123 | KM652006 | KM652045 |  |
| Hirsutella satumaensis | ARSEF 996 | Lepidoptera: Pyralidae | KM652082 | KM652125 | KM652008 | KM652047 |  |
| *Hirsutella strigosa* | ARSEF 2197 | Hemiptera: Delphacidae | KM652085 | KM652129 | KM652012 | KM652050 |  |
| *Hirsutella subulata* | ARSEF 2227 | Lepidoptera: Microlepidoptea | KM652086 | KM652130 | KM652013 | KM652051 |  |
| *Hirsutella thompsonii* | ARSEF 257 | Acari: Eriophyidae | KM652091 | KM652136 | KM652019 | KM652054 |  |
| Hirsutella thompsonii | ARSEF 254 | Acari: Eriophyidae | KM652101 | KM652149 | KM652028 | KM652062 |  |
| *Ophiocordyceps acicularis* | OSC 110987 | Coleoptera larva | EF468950 | EF468805 | EF468744 | EF468852 |  |
| *Ophiocordyceps agriotidis* | ARSEF 5692 | Coleoptera larva | DQ522540 | DQ518754 | DQ522322 | DQ522368 | DQ522418 |
| ***Ophiocordyceps albastroma*** | YFCC 15079242 | Hepialidae larva | **OQ626658** | **OQ629823** | **OQ622098** | **OQ622104** | **-** |
| ***Ophiocordyceps albastroma*** | YFCC 15079243 | Hepialidae larva | **OQ626659** | **OQ629824** | **OQ622099** | **OQ622105** | **OQ622110** |
| *Ophiocordyceps appendiculata* | NBRC 106960 | Coleoptera (larva) | JN941728 | JN941413 | AB968577 | JN992462 | AB968539 |
| *Ophiocordyceps arborescens* | NBRC 105890 | Moth larvae (*Cossida* sp.; Cossidae; Lepidoptera) | AB968387 | AB968415 | AB968573 |  | AB968535 |
| *Ophiocordyceps barnesii* | BCC 28560 | Coleoptera larva | EU408776 |  |  | EU408773 | EU418599 |
| *Ophiocordyceps blattae* | BCC 34765 | Blattodea |  |  | MT533484 | MT533478 |  |
| *Ophiocordyceps brunneinigra* | TBRC 8093 | Hemiptera |  | MF614654 | MF614638 | MF614668 | MF614681 |
| *Ophiocordyceps brunneiperitheciata* | TBRC 8099 | Lepidoptera larva |  | MF614659 | MF614644 |  | MF614684 |
| *Ophiocordyceps clavata* | NBRC 106962 | Coleopteran larva | JN941726 | JN941415 | AB968587 | JN992460 | AB968548 |
| *Ophiocordyceps communis* | BCC 1842 | Termitidae (adult termite) |  | MH753680 | MK284266 | MK214110 | MK214096 |
| *Ophiocordyceps crinalis* | GDGM 17327 | Lepidoptera larva | KF226253 | KF226254 | KF226256 | KF226255 |  |
| *Ophiocordyceps entomorrhiza* | KEW 53484 | Coleopteran larva | EF468954 |  | EF468749 | EF468857 |  |
| *Ophiocordyceps formosana* | TNM F13893 | Coleoptera | KJ878908 |  | KJ878956 | KJ878988 | KJ878943 |
| *Ophiocordyceps geometridicola* | TBRC 8094 | Lepidoptera larva |  | MF614647 | MF614631 | MF614664 | MF614678 |
| *Ophiocordyceps irangiensis* | NBRC 101400 | Hymenoptera (adult ant) |  | JN941426 |  | JN992449 |  |
| *Ophiocordyceps iranginensis* | BCC 82795 | Hymenoptera (*Polyrhachis illaudata*) |  |  | MH028186 | MH028164 | MH028174 |
| *Ophiocordyceps karstii* | MFLU 15-3884 | Lepidoptera (Hepialus jianchuanensis) | KU854952 |  | KU854945 | KU854943 |  |
| *Ophiocordyceps khonkaenensis* | BCC81462^T^ | Hemiptera (cicada nymph) | MK632126 |  | MK632075 | MK632168 | MK632157 |
| *Ophiocordyceps khonkaenensis* | BCC81464 | Hemiptera (cicada nymph) | MK632128 | MK632103 | MK632077 | MK632170 | MK632159 |
| *Ophiocordyceps kimflemingiae* | SC09B | Hymenoptera (Camponotus castaneus/americanus） | KX713631 | KX713620 | KX713698 | KX713724 |  |
| *Ophiocordyceps konnoana* | EFCC 7315 | Coleoptera | EF468959 |  | EF468753 | EF468861 | EF468916 |
| *Ophiocordyceps lanpingensis* | YHOS0705 | Hepialidae larva | KC417458 | KC417460 | KC417462 | KC417464 | KC456333 |
| *Ophiocordyceps lanpingensis* | YHOS0707 | Hepialidae larva | KC417459 | KC417461 | KC417463 | KC417465 |  |
| *Ophiocordyceps liangshanensis* | YFCC 8577 | Lepidoptera (Hepialidae larva) | MT774218 | MT774225 | MT774246 | MT774232 | MT774239 |
| *Ophiocordyceps liangshanensis* | YFCC 15099244 | Lepidoptera (Hepialidae larva) | OQ608672 | OQ608804 | OQ622100 | OQ622106 | OQ622111 |
| *Ophiocordyceps longissima* | EFCC 6814 | Hemiptera (cicada nymph) |  | EF468817 | EF468757 | EF468865 |  |
| *Ophiocordyceps longissima* | NBRC 106965 | Hemiptera: Cicadidae (cicada nymph) | AB968392 | AB968420 | AB968584 |  | AB968546 |
| *Ophiocordyceps longistromata* | BCC 44497 | Lepidoptera larva |  | MT118178 | MT118170 |  | MT118191 |
| *Ophiocordyceps macroacicularis* | NBRC 105889 | Lepidoptera larva | AB968390 | AB968418 | AB968576 |  | AB968538 |
| *Ophiocordyceps macroacicularis* | NBRC 100685 | Lepidoptera larva | AB968388 | AB968416 | AB968574 |  | AB968536 |
| *Ophiocordyceps multiperitheciata* | BCC 22861 | Lepidoptera larva |  | MF614656 | MF614640 | MF614670 | MF614683 |
| *Ophiocordyceps myrmecophila* | TNS 27120 | Hymenoptera (Adult ant) | KJ878929 | KJ878895 | KJ878975 | KJ879009 |  |
| *Ophiocordyceps nigra* | TNS 16252 | Hemiptera | KJ878941 | KJ878906 | KJ878986 |  |  |
| *Ophiocordyceps nigrella* | EFCC 9247 | Lepidoptera larva | EF468963 | EF468818 | EF468758 | EF468866 | EF468920 |
| ***Ophiocordyceps nigristroma*** | YHH 1408002 | Hepialidae larva | **-** | **-** | **OQ622101** | **OQ622107** | **-** |
| ***Ophiocordyceps nigristroma*** | YFCC 17059245 | Hepialidae larva | **OQ629822** | **OQ629825** | **OQ622102** | **OQ622108** | **OQ622112** |
| *Ophiocordyceps nujiangensis* | YFCC 8880 | Hepialidae (larvae) | ON723384 | ON723381 | ON868820 | ON868823 | ON868826 |
| *Ophiocordyceps nujiangensis* | YFCC 8894 | Hepialidae (larvae) |  | ON723382 | ON868821 | ON868824 |  |
| *Ophiocordyceps pauciovoperitheciata* | TBRC 8096 | Lepidoptera larva |  | MF614649 | MF614636 | MF614665 | MF614672 |
| *Ophiocordyceps pingbianensis* | YFCC 8075 | Coleoptera (beetle larva) |  |  |  |  |  |
| *Ophiocordyceps pruinosa* | NHJ 12994 | Hemiptera | EU369106 | EU369041 | EU369024 | EU369063 | EU369084 |
| *Ophiocordyceps pulvinata* | TNS F30044 | Hymenoptera (*Camponotus obscuripes*) | GU904208 |  | GU904209 | GU904210 |  |
| *Ophiocordyceps ravenelii* | OSC 110995 | Coleoptera (beetle larva) | DQ522550 | DQ518764 | DQ522334 | DQ522379 | DQ522430 |
| *Ophiocordyceps rhizoidea* | NHJ 12522 | Termite (Isoptera) | EF468970 | EF468825 | EF468764 | EF468873 | EF468923 |
| *Ophiocordyceps rhizoidea* | NHJ 12529 | Termite (Isoptera) | EF468969 | EF468824 | EF468765 | EF468872 | EF468922 |
| *Ophiocordyceps robertsii* | KEW 27083 | Lepidoptera |  | EF468826 | EF468766 |  |  |
| *Ophiocordyceps rubiginosiperitheciata* | NBRC 106966 | Coleoptera (larva) | JN941704 | JN941437 | AB968582 | JN992438 | AB968544 |
| *Ophiocordyceps salganeicola* | Mori01 | Blattodea (Blattoidea) | MT741705 | MT741719 | MT759575 | MT759578 | MT759580 |
| *Ophiocordyceps satoi* | J7 | Hymenoptera (Polyrhachis lamellidens) | KX713653 | KX713599 | KX713683 | KX713711 |  |
| *Ophiocordyceps sinensis* | EFCC 7287 | Lepidoptera pupa | EF468971 | EF468827 | EF468767 | EF468874 | EF468924 |
| *Ophiocordyceps sinensis* | YHH 1805 | Lepidoptera (Hepialidae larva) | MK984568 | MK984580 | MK984572 | MK984587 | MK984576 |
| *Ophiocordyceps sobolifera* | TNS F18521 | Hemiptera: Cicadidae | KJ878933 | KJ878898 | KJ878979 | KJ879013 |  |
| *Ophiocordyceps sobolifera* | NBRC 106967 | Hemiptera (cicada nymph) | AB968395 | AB968422 | AB968590 |  |  |
| *Ophiocordyceps spataforae* | OSC 128575 | Hemiptera adult | EF469126 | EF469079 | EF469064 | EF469093 | EF469110 |
| *Ophiocordyceps sphecocephala* | NBRC 101753 | Hymenoptera (adult wasp) | JN941695 | JN941446 | AB968592 | JN992429 | AB968553 |
| *Ophiocordyceps sphecocephala* | NBRC 101752 | Hymenoptera (adult wasp) | JN941696 | JN941445 | AB968591 | JN992430 | AB968552 |
| *Ophiocordyceps stylophora* | OSC 110999 | Coleopteran larva | EF468982 | EF468837 | EF468777 | EF468882 | EF468931 |
| *Ophiocordyceps unilateralis* | OSC 128574 | Hymenoptera | DQ522554 | DQ518768 | DQ522339 | DQ522385 | DQ522436 |
| *Ophiocordyceps unituberculata* | YFCC HU1301 | Lepidoptera larva | KY923214 |  | KY923216 | KY923218 | KY923220 |
| *Ophiocordyceps xuefengensis* | GZUH2012HN13 | *Phassus nodus* larvae | KC631787 |  | KC631792 | KC631797 |  |
| *Ophiocordyceps xuefengensis* | YFCC 16049246 | *Phassus nodus* larvae | OQ608721 | OQ621604 | OQ622103 | OQ622109 | OQ622113 |
| *Ophiocordyceps yakusimensis* | HMAS 199604 | Hemiptera (cicada nymph) | KJ878938 | KJ878902 |  | KJ879018 | KJ878953 |
| *Paraisaria amazonica* | Ophama2026 | Orthoptera Acrididae (imago) | KJ917562 | KJ917571 | KM411989 | KP212902 | KM411982 |
| *Paraisaria blattarioides* | Ophbla014 | Dyctioptera Blattodea (imago) | KJ917559 | KJ917570 | KM411992 | KP212910 |  |
| *Paraisaria gracilis* | EFCC 8572 | Coleoptera Elateridae (larva) | EF468956 | EF468811 | EF468751 | EF468859 | EF468912 |
| *Paraisaria gracilis* | EFCC 3101 | Coleoptera Elateridae (larva) | EF468955 | EF468810 | EF468750 | EF468858 | EF468913 |
| *Paraisaria orthopterorum* | BBC 88305 | Orthoptera (nymph) |  | MK332583 | MK214080 | MK214084 |  |
| *Paraisaria orthopterorum* | TBRC 9710 | Orthoptera (nymph) |  | MK332582 | MK214081 | MK214085 |  |
| *Paraisaria yodhathaii* | BBH 43163 | Coleoptera Elateridae (larva) |  | MK332584 | MH211353 | MH211349 |  |
| *Tolypocladium capitatum* | NBRC 106327 | *Elaphomyces* sp. | JN941737 | JN941404 |  | JN992471 |  |
| *Tolypocladium japonicum* | OSC 110991 | *Elaphomyces* sp. | DQ522547 | DQ518761 | DQ522330 | DQ522375 | DQ522428 |
| *Tolypocladium inflatum* | OSC 71235 | Coleoptera larva | EF469124 | EF469077 | EF469061 | EF469090 | EF469108 |
| [*Tolypocladium ophioglossoides*](https://www.ncbi.nlm.nih.gov/Taxonomy/Browser/wwwtax.cgi?id=71617) | CBS 100239 | Elaphomyces sp. | KJ878910 | KJ878874 | KJ878958 | KJ878990 | KJ878944 |
