# Supplementary material for: ﻿Mitogenomics, phylogeny and morphology reveal two new entomopathogenic species of Ophiocordyceps (Ophiocordycipitaceae, Hypocreales) from south-western China
Source: MycoKeys. 2024 Sep 26;109:49–72. doi: 10.3897/mycokeys.109.124975 (PMC11450462; doi:10.3897/mycokeys.109.124975)
Supplement: Supplementary material 1 — Supplementary information [file mycokeys-109-049-s001.zip › revised Supplementary Files/Table S3.docx]

**Table S3 The information of species and their mitochondrial genomes for constructing the mitochondrial-genome phylogenetic tree of Hypocreales**

| **Order** | **Family** | **Species** | **Genbank ID** | **Strain** | **Notes** |
| --- | --- | --- | --- | --- | --- |
| Eurotiales | Aspergillaceae | *Penicillium citrinum* | NC047444 | JH1205 | Outgroup |
| Sordariales | Sordariaceae | *Neurospora crassa* | KY498478 | FGSC 9718 |  |
| Hypocreales | Bionectriaceae | *Acremonium chrysogenum* | NC023268 | ATCC:11550 |  |
|  |  | *Acremonium fuci* | NC029851 | 3a34 |  |
|  |  | *Clonostachys compactiuscula* | MW030498 | YFCC 897 |  |
|  |  | *Clonostachys rogersoniana* | MW030499 | YFCC 899 |  |
|  |  | *Clonostachys rosea* | NC036667 | 6792 |  |
|  | Clavicipitaceae | *Epichloe festucae* | NC032064 | AR5 |  |
|  |  | *Epichloe hybrida* | KX066187 | Lp1 |  |
|  |  | *Epichloe typhina* | NC032063 | E8 |  |
|  |  | *Metacordyceps chlamydosporia* | NC022835 | 170 |  |
|  |  | *Metarhizium album* | MW448543 | ARSEF1941 |  |
|  |  | *Metarhizium brunneum* | LR792747 | ARSEF 4556 |  |
|  |  | *Metarhizium rileyi* | MT107156 | RCEF 4871 |  |
|  |  | *Metarhizium robertsii* | JELW01000367 | ARSEF 2575 |  |
|  |  | *Orbiocrella petchii* | MT447058 | SD3 |  |
|  | Cordycipitaceae | *Akanthomyces lecanii* | NC046840 | RCEF1005 |  |
|  |  | *Beauveria bassiana* | NC010652 | Bb13 |  |
|  |  | *Beauveria brongniartii* | NC011194 | IMBST95031 |  |
|  |  | *Beauveria caledonica* | NC030636 | fhr1 |  |
|  |  | *Beauveria malawiensis* | NC030635 | k89 |  |
|  |  | *Beauveria pseudobassiana* | NC022708 | C1010 |  |
|  |  | *Cordyceps chanhua* | MH734937 | JGS-7 |  |
|  |  | *Cordyceps cicadae* | NC041489 | CCAD02 |  |
|  |  | *Cordyceps farinosa* | OM201302 | YFCC8744 |  |
|  |  | *Cordyceps militaris* | NC022834 | EFCC-C2 |  |
|  |  | *Cordyceps pruinosa* | MN515031 | CP1 |  |
|  |  | *Cordyceps tenuipes* | MK234910 | YFCC 2017002 |  |
|  |  | *Lecanicillium saksenae* | NC028330 | CGMCC5329 |  |
|  |  | *Parengyodontium album* | KX061492 | ATCC:56482 |  |
|  |  | *Samsoniella hepiali* | KJ764671 | — |  |
|  | Hypocreaceae | *Hypomyces aurantius* | NC030206 | — |  |
|  |  | *Paecilomyces penicillatus* | NC043850 | SAAS_ppe1 |  |
|  |  | *Trichoderma asperellum* | NC037075 | B05 |  |
|  |  | *Trichoderma hamatum* | NC036144 | — |  |
|  |  | *Trichoderma reesei* | NC003388 | QM9414 |  |
|  | Nectreaceae | *Fusarium commune* | NC036106 | JCM11502 |  |
|  |  | *Fusarium culmorum* | NC026993 | CBS 139512 |  |
|  |  | *Fusarium fujikuroi* | JX910420 | IMI58289 |  |
|  |  | *Fusarium gerlachii* | NC025928 | CBS 123666 |  |
|  |  | *Fusarium oxysporum* | NC017930 | F11 |  |
|  |  | *Fusarium proliferatum* | LT841261 | ITEM2400 |  |
|  |  | *Fusarium solani* | NC016680 | mpVI |  |
|  |  | *Fusarium verticillioides* | NC016687 | 7600 |  |
|  |  | *Ilyonectria destructans* | NC030340 | 2007/P/476 |  |
|  | Ophiocordycipitaceae | *Hirsutella minnesotensis* | NC027660 | 3608 |  |
|  |  | *Hirsutella rhossiliensis* | NC030164 | USA-87-5 |  |
|  |  | *Hirsutella thompsonii* | NC040165 | ARSEF 9457 |  |
|  |  | *Hirsutella vermicola* | NC036610 | AS3.7877 |  |
|  |  | ***Ophiocordyceps albastroma*** | **OQ658681** | **YFCC 15079243** | Material examined |
|  |  | *Ophiocordyceps liangshanensis* | OQ658679 | YFCC 15099244 |  |
|  |  | ***Ophiocordyceps nigristroma*** | **OQ658680** | **YFCC 17059245** | Material examined |
|  |  | *Ophiocordyceps pingbianensis* | MW042690 | YFCC 8075 |  |
|  |  | *Ophiocordyceps sinensis* | NC034659 | CCTCC AF 2017003 |  |
|  |  | *Ophiocordyceps xuefengensis* | OQ658678 | YFCC 16049246 |  |
|  |  | *Tolypocladium cylindrosporum* | NC046839 | ARSEF 963 |  |
|  |  | *Tolypocladium inflatum* | KY924883 | ARSEF 616 |  |
|  |  | *Tolypocladium guangdongense* | NC054274 | GD15 |  |
|  |  | *Tolypocladium ophioglossoides* | NC031384 | L2 |  |
